# Generation of Red-Shifted Cameleons for Imaging Ca^2+^ Dynamics of the Endoplasmic Reticulum

**DOI:** 10.3390/s150613052

**Published:** 2015-06-04

**Authors:** Markus Waldeck-Weiermair, Helmut Bischof, Sandra Blass, Andras T. Deak, Christiane Klec, Thomas Graier, Clara Roller, Rene Rost, Emrah Eroglu, Benjamin Gottschalk, Nicole A. Hofmann, Wolfgang F. Graier, Roland Malli

**Affiliations:** Institute of Molecular Biology and Biochemistry, Centre of Molecular Medicine, Medical University of Graz, Harrachgasse 21, 8010 Graz, Austria; E-Mails: bischof.helmut@gmail.com (H.B.); sandra.blass@medunigraz.at (S.B.); andras.deak@medunigraz.at (A.T.D.); christiane.klec@medunigraz.at (C.K.); thomas.graier@medunigraz.at (T.G.); clara.roller@gmx.at (C.R.); rene.rost@medunigraz.at (R.R.); emrah.eroglu@medunigraz.at (E.E.); benjamin.gottschalk@medunigraz.at (B.G.); nicole.hofmann@medunigraz.at (N.A.H.); wolfgang.graier@medunigraz.at (W.F.G.); roland.malli@medunigraz.at (R.M.)

**Keywords:** Förster resonance energy transfer, FRET, calcium, endoplasmic reticulum, store operated calcium entry, SOCE, clover, mRuby2, dissociation constant

## Abstract

Cameleons are sophisticated genetically encoded fluorescent probes that allow quantifying cellular Ca^2+^ signals. The probes are based on Förster resonance energy transfer (FRET) between terminally located fluorescent proteins (FPs), which move together upon binding of Ca^2+^ to the central calmodulin myosin light chain kinase M13 domain. Most of the available cameleons consist of cyan and yellow FPs (CFP and YFP) as the FRET pair. However, red-shifted versions with green and orange or red FPs (GFP, OFP, RFP) have some advantages such as less phototoxicity and minimal spectral overlay with autofluorescence of cells and fura-2, a prominent chemical Ca^2+^ indicator. While GFP/OFP- or GFP/RFP-based cameleons have been successfully used to study cytosolic and mitochondrial Ca^2+^ signals, red-shifted cameleons to visualize Ca^2+^ dynamics of the endoplasmic reticulum (ER) have not been developed so far. In this study, we generated and tested several ER targeted red-shifted cameleons. Our results show that GFP/OFP-based cameleons due to miss-targeting and their high Ca^2+^ binding affinity are inappropriate to record ER Ca^2+^ signals. However, ER targeted GFP/RFP-based probes were suitable to sense ER Ca^2+^ in a reliable manner. With this study we increased the palette of cameleons for visualizing Ca^2+^ dynamics within the main intracellular Ca^2+^ store.

## 1. Introduction

The endoplasmic reticulum (ER) exhibits a 1000-fold higher concentration of Ca^2+^ than that in the cytosol or mitochondria by storing more than 90% of intracellular Ca^2+^ in less than 10% of total cell volume [1,2]. The release of Ca^2+^ from this internal Ca^2+^ store is important for multiple signaling events [3] that regulate ATP production [4], insulin secretion [5], muscle contraction [6,7] or gene regulation [8,9]. Based on the importance of the ER Ca^2+^ content for cell signaling, processes that refill the ER with Ca^2+^ are necessary. Particularly, in non-excitable cells, the release of Ca^2+^ from the ER induces the so-called store operated Ca^2+^ entry (SOCE). SOCE is based on the interaction of the stromal interacting molecule 1 (STIM1) with the Ca^2+^ channel pore-forming unit ORAI1 in the plasma membrane [10]. The fundamental role of SOCE is to restore Ca^2+^ of the ER during and after cell stimulation. Interestingly, in endothelial cells mitochondria are important to direct entering Ca^2+^ to the ER during the process of ER Ca^2+^ refilling [11,12,13]. In addition to the importance of ER Ca^2+^ for the generation of global and local Ca^2+^ signals, the ER Ca^2+^ content considerably impacts the protein folding machineries within this organelle [14]. A loss of Ca^2+^ within the ER is associated with the induction of the unfolding protein response (UPR), which can also lead to cell death [15]. Recently, it was shown that the energy supply of the ER is tightly coupled to the organelles’ Ca^2+^ homeostasis. In order to investigate the dynamic storage of Ca^2+^ by the ER and to correlate changes of the ER Ca^2+^ content with other ER-related signaling events under physiological as well as pathophysiological conditions, the demand on reliable ER Ca^2+^ sensors is obvious.

Although some chemical indicators have been successfully established for assessing the ER Ca^2+^ homeostasis [13,16], these requirements were better realized by sophisticated genetically encoded Ca^2+^ indicators (GECIs). In general, GECIs can be categorized into two classes: the FRET based cameleons that consists of two fluorescent proteins (FP) and the single FP-based GCaMP-like sensors [17,18]. Both types take different advantages, e.g., the cameleons benefit from ratiometric signal changes, photo- and pH-stability [19,20], whereas single FP-based indicators have larger signal changes, better dynamic kinetics and they are more variable in their fluorescent hues [21,22,23,24,25]. Cameleon-type indicators are constructed of a genetic fusion of calmodulin (CaM) and a small Ca^2+^-CaM binding peptide (M13), which is flanked by the donor and acceptor fluorescent proteins. The Ca^2+^-dependent interaction of CaM with M13 induces a conformational change that narrows the distance of the two FPs and, thus, results in an increase of the FRET ratio signal [26] In contrast, in GCaMP-type indicators a circular permutated FP (cpFP) is flanked by M13 at its N-terminus and CaM at its C-terminus. Herein, the interaction of the Ca^2+^ dependent motifs changes the environment of the cpFP’s chromophore inducing an intensiometric increase of the fluorescence [27]. Moreover, the cameleons are more accessible to genetically shift the balance of the dissociation constant (*K*_d_) [28,29], but, due to the presence of two different FPs, the possibilities for multi-color imaging are limited [30,31]. For measuring Ca^2+^ signals of the ER, both classes of GECIs have been conjugated with a calreticulin signal [32] and the classical ER retention sequence [33] which target the probes into the ER lumen.

However, so far most of GECIs were used to detect Ca^2+^ in the cytosol or mitochondria. By mutating the original CaM-M13 domain in order to reduce the Ca^2+^ binding affinity the first ER Ca^2+^ cameleons were developed. So far a cameleon named D1ER represents the state-of-the-art probe for sensing [Ca^2+^]_ER_ [34] This probe consists of an enhanced CFP (ECFP) as FRET donor and the yellow FP Citrine as FRET acceptor flanking the Ca^2+^ sensitive motif D1 (design 1). In D1 the CaM and M13 domains were redesigned to optimize the Ca^2+^ affinity for quantifying ER Ca^2+^ dynamics.

In this study, we engineered numerous red-shifted ER-targeted cameleons based on the optimized D1 motif. Such bathochromic probes can be better used for simultaneous measurements of [Ca^2+^]_ER_ and [Ca^2+^]_Cyto_ by combining these novel GECIs with fura-2. Our results show that despite an ER targeting and ER retention sequences, cameleons containing cpEGFP on the C-terminus show poor ER localization. Moreover, the GFP/OFP-based ER targeted cameleons gained Ca^2+^ sensitivity, which make these probes impractical for sensing ER Ca^2+^ signals. Finally, we found suitable GFP/RFP FRET pairs for the development of functional ER targeted cameleons, which are suitable for dual recordings of [Ca^2+^]_ER_ and [Ca^2+^]_Cyto_ in single individual cells.

## 2. Experimental Section

### 2.1. Materials

For culturing cells, RPMI-1640, fetal calf serum (FCS), penicillin, streptomycin, sodium pyruvate, vitamins, essential amino acids and all plastic ware were obtained from PAA laboratories (Pasching, Austria). Dulbecco’s modified eagle’s medium (DMEM), histamine dihydrochloride (histamine), 2,5-di-t-butyl-1,4-benzohydroquinone (BHQ) and digitonin were purchased from Sigma-Aldrich (Vienna, Austria). Ionomycin (free acid) was delivered from abcamBiochemicals (Cambridge, UK), fura-2/AM (Fura2) from Teflabs (Texas Fluorescence Labs Inc., Austin, TX, USA) and T_4_ DNA Ligase from Thermo Scientific (Braunschweig, Germany). TagRFP and Camui-CR plasmids were purchased from Addgene. GoTaq^®^ Hot Start Polymerase, restriction enzymes, Wizard^®^ SV gel and PCR Clean-up System, PureYield^TM^ Plasmid Maxiprep System and Transfast^TM^ transfection reagent were obtained from Promega (Mannheim, Germany). All other materials and chemicals were from Roth (Karlsruhe, Germany).

### 2.2. Engineering of Sensors

For plasmid construction of the various genetically encoded Ca^2+^ indicators (GECIs) a pcDNA3.1(-) vector containing the coding sequences (cds) of the recently published cameleons D1GO-Cam or mtD1GO-Cam were used as templates. Substitution of the cds from the different fluorescent proteins (FP) was done by PCR amplification, restriction digestion and ligation to the N- or C-terminal end of the sensor sequence. Targeting of cameleons to the ER was achieved by primer annealing and subsequent ligation of the ER targeting sequence from calreticulin to the N-terminal end of the GECIs. For proper ER retention, the coding sequence lysine-aspartic acid-glutamic acid-leucine (KDEL) was added to the reverse primers of the C-terminal inserted fluorescent protein sequence. A list of all primers and restriction enzymes used is given in Table S1.

### 2.3. Cell Culture and Transfection

Human embryonic kidney 293 stably expressing ryanodine receptor 2 (HEK E2) [35] and HeLa cells (HeLa) [36] were cultured in DMEM supplemented with 10% FCS, 100 U/mL penicillin, 100 µg/mL streptomycin. INS-1 832/13 cells (INS-1) [5] kindly obtained from C. B. Newgard (Duke University School of Medicine, Durham, NC, USA) were cultured with RPMI-1640 medium containing 10% FCS, 11.1 mM D-glucose, 1 mM sodium pyruvate, 5 µM mercaptoethanol, 100 U/mL penicillin and 100 µg/mL streptomycin. The 3 different cell lines were maintained in a cell incubator at 37 °C in a humidified atmosphere with 5% CO_2_ and 95% air. For transfection and experiments cells were cultured on glass cover slips (Ø = 30 mm). Transfection was performed at 50%–80% cell confluence by adding a transfection mixture of DMEM supplemented with the respective sensor plasmid DNA and Transfast^TM^ transfection reagent. After 12–18 h incubation, transfection mixture was replaced by normal culture medium. Imaging experiments were performed 40–50 h after transfection.

### 2.4. Fura-2 Loading and Experimental Buffers for Ca^2+^ Recordings

For dual recordings of [Ca^2+^]_Cyto_ and [Ca^2+^]_ER_ cells expressing one of the red-shifted cameleons were loaded with 3 µM fura-2/AM at room temperature for 30–40 min in a buffer composed of (in mM): 2 CaCl_2_ 135 NaCl, 5 KCl, 1 MgCl_2_, 1 HEPES, 2.6 NaHCO_3_, 0.44 KH_2_PO_4_, 0.34 Na_2_HPO_4_, 10 D-glucose, 0.1% vitamins, 0.2% essential amino acids and 1% penicillin/streptomycin pH 7.4. Fura-2 loaded cells as wells as non-loaded cells were washed and stored in this buffer prior to imaging experiments. For Ca^2+^ measurements, cells were perfused in a HEPES buffered solution containing (in mM): 140 NaCl, 5 KCl, 1 MgCl_2_, 1 HEPES and 10 D-Glucose pH 7.4 (0 mM Ca^2+^). Additionally, either 1 mM EGTA (EGTA), 1 mM CaCl_2_ (1 mM Ca^2+^) or 2 mM CaCl_2_ (2 mM Ca^2+^) were added to 0 mM Ca^2+^ and pH adjusted to 7.4.

### 2.5. Spectral Scans and K_d_ Determination

HeLa cells transfected with ER targeted cameleons were permeabilized with 5 µM digitonin and 10 µM ionomycin (digitonin/ionomycin) in 2 mM Ca^2+^ buffer for 3 min. For spectral scans, EGTA buffer supplemented with digitonin/ionomycin was added for another 3 min to obtain maximum depletion of Ca^2+^ from the ER. Individual red-shifted ER sensors were alternately exposed at 400 to 490 nm in 10 nm steps for 400 ms each through the whole experimental time course. Emitting light of FRET donor and FRET acceptor was recorded at 510 and 560 nm, respectively, as previously described [37] Signal to noise ratios were then calculated at all excitation wavelengths for each sensor. Dissociation constant (*K*_d_) of [Ca^2+^] from D1ERCmR2 was determined *in situ*. The ER of permeabilized HeLa cells was depleted using a 3 mM EGTA buffer. Free Ca^2+^ concentrations were calculated using the CaBuff software (G. Droogmans, Fysiologie, Leuven) and appropriate amount of Ca^2+^ were added to the 3 mM EGTA buffer to obtain buffer solutions of 1, 3, 10, 30, 100, 300, 1000, 2000 and 10,000 µM free Ca^2+^.

### 2.6. Ca^2+^ Recordings and Data Acquisition

Ca^2+^ imaging was done using the TiLL iMIC (Till Photonics, Graefelfing, Germany) digital wide field imaging system, as described previously [12,31,38,39]. Dual Ca^2+^ recording of fura-2 and red shifted cameleons was performed by alternated excitations at 340, 380 and 480 nm and emissions were captured at 510 and 560 nm. D1ER was excited at 430 nm and emission was collected using the dichrotome dual emission filterset (dichroic 535dcxr, CFP emitter 482/18 nm and YFP emitter 535/3 nm). Data acquisition and control of the digital fluorescence microscope was done using the live acquisition software version 2.0.0.12 (Till Photonics). Results of FRET measurements are either shown as the ratio of (F_535_/F_480_)/R_0_ for D1ER or (F_560_/F_510_)/R_0_ for red-shifted cameleons.

### 2.7. Confocal Imaging

Images of subcellular structures for colocalization were taken from cells coexpressing D1ER and either D1ERGO-Cam1 or D1ERGO-Cam2. Fluorescence of D1ERCmR2 expressing cells was either imaged alone or together after fura-2/AM loading. All images were recorded with an array confocal laser scanning microscope (ACLSM) built on a fully automatic inverse microscope (Axio Observer.Z1, Zeiss, Göttingen, Germany) equipped with VoxCell Scan^®^ (VisiTech, Visitron Systems) using a 100 × objective (Plan-Fluor 100 × /1.45 oil, Zeiss), as described previously [12,37,40]. Excitation was done using laser light of diode lasers (Visitron Systems): Fura-2 was excited at 405 nm (120 mW), CFP of D1ER was excited at 445 nm (50 mW), Clover of D1ERCmR2 was excited at 473 nm (50 mW), mKO and mRuby2 of D1ERGO-Cam1, D1ERGO-Cam2 and D1ERCmR2 were excited at 515 nm (50 mW). Emitted light was acquired with emission filters ET460/50m for fura-2 (DAPI filter), ET480/40m for CFP, ET525/50m for Clover and E570LPv2 for mKO and mRuby2 (Chroma Technologies, Corporation, VT, USA). A Photometrics CCD camera (CoolSnap HQ2) was used to capture all images. Quantitative ER colocalization computations were performed with the integrated morphometric analysis plug-in of MetaMorph 7.7.0.0 software (Visitron).

### 2.8. Statistics

Statistical relevant data are shown as means ± SEM, where *n* represents the number of cells. Analyses were done using unpaired Student’s *t test* and evaluation of significance was considered to be *p <* 0.05.

## 3. Results and Discussion

### 3.1. GFP/OFP FRET-Based Cameleons are Unsuitable to Measure Ca^2+^ Dynamics of the ER

**Figure 1:**
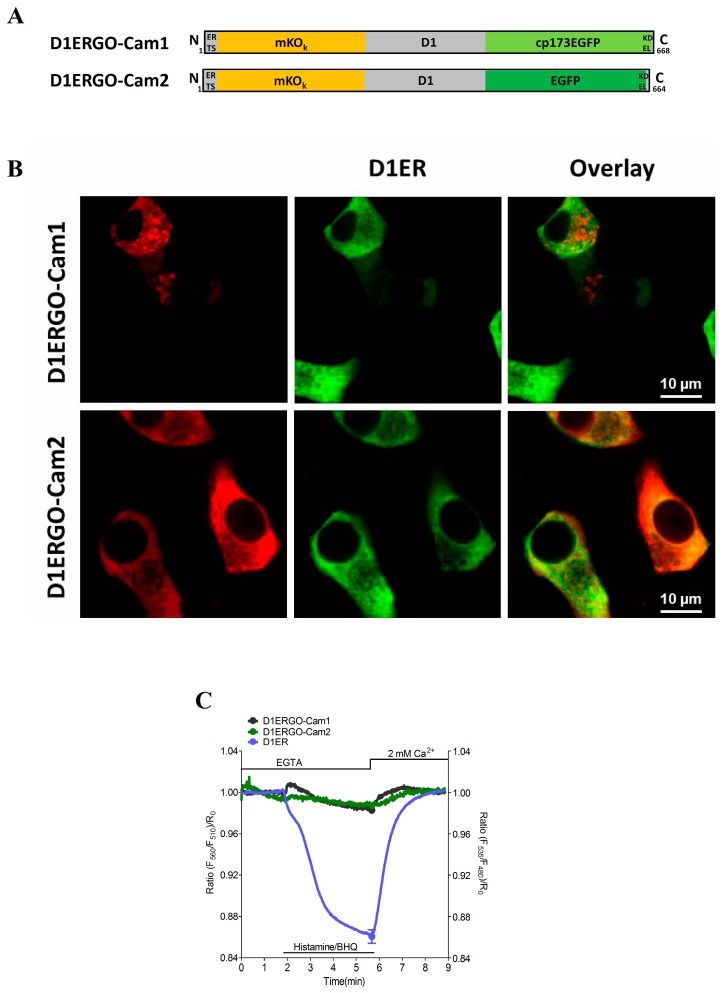
Evaluation of GFP/OFP cameleons upon ER targeting: (**A**) schematic composition of D1ERGO-Cam1 and D1ERGO-Cam2; and (**B**) representative confocal images for colocalization analysis. INS-1 cells were co-transfected with D1ER and either D1ERGO-Cam1 or D1ERGO-Cam2, respectively. Images of GFP/OFP-based cameleons were taken from the mKO emission at 570 nm (left panels); D1ER was monitored in its CFP emission at 480 nm (middle panels); and an overlay of images (right panels); (**C**) Average curves of FRET measurements in HeLa cells expressing either D1ER (cyan curve, n = 14), D1ERGO-Cam1 (dark grey curve, n = 10) or D1ERGO-Cam2 (green curve, n = 7) upon treatment with 100 µM histamine and 15 µM BHQ in the absence of extracellular Ca^2+^. SOCE was accomplished by the subsequent addition of 2 mM Ca^2+^.

Analogous to recently developed red-shifted cameleons for imaging Ca^2+^ signals either within the cytosol (D1GO-Cam) or mitochondria (mtD1GO-Cam) [31] we constructed the ER targeted Ca^2+^ probe D1ERGO-Cam1 and D1ERGO-Cam2 (Figure 1A). For ER targeting the respective red-shifted cameleon was fused with the targeting sequence of calreticulin on the N-terminus and a KDEL retention sequence was added at the C-terminus, respectively. However, ER localization of D1ERGO-Cam1 was poor in the rat pancreatic beta cell line, INS-1 (Figure 1B). Colocalization analysis revealed that only 23.46% ± 1.89% (n = 10) of the D1ERGO-Cam1 fluorescence merged with the D1ER signal in this cell type. The localization of D1ERGO-Cam1 in non-ER compartments might be due to the circularly permutated enhanced green fluorescent protein (cpEGFP) on the C-terminus, as it has been reported that circularly permutated Venus is not localized well in the ER [17]. Hence, we speculate that circularly permutated FPs mask the ER retention sequence, so that these constructs are exported from the organelle. Indeed, the exchange of cpEGFP by EGFP in D1ERGO-Cam2 (Figure 1A) considerably improved the ER targeting of the red-shifted cameleon (Figure 1B). D1ERGO-Cam2 co-localized by 83.34% ± 2.33% (n = 10) with D1ER in INS-1 cells. However, both red-shifted cameleons D1ERGO-Cam1 and D1ERGO-Cam2 containing cpEGFP or EGFP as the FRET donor and the monomer Kusabira Orange (mKO) as the FRET acceptor hardly sensed Ca^2+^ dynamics within the ER (Figure 1C). The poor performance of these red-shifted probes to report changes in ER Ca^2+^ is most likely due to an increased sensitivity of the cameleons to bind Ca^2+^. In a recent study we demonstrated that despite the reported dissociation constant (*K*_d_) of approximately 200 µM of the D1 domain in D1ER, exchanging the CFP/YFP FRET pair by GFP/OFP and removing the ER targeting sequence resulted in a shift of the *K*_d_ to 1 µM [31] Accordingly, these red-shifted D1 cameleons work well in the cytosol and mitochondria. In this study, we targeted the GFP/OFP-based D1 cameleons in the ER lumen in order to test if the localization within this organelle impacts the Ca^2+^ binding affinity of the probe. Our results, however, confirmed that independent of the subcellular localization GFP/OFP-based D1 cameleons have a significantly increased Ca^2+^ sensitivity. Next we constructed numerous other red-shifted ER targeted cameleons that might have lower sensitivities and, hence, suitable for visualizing dynamic changes of [Ca^2+^]_ER_.

### 3.2. Development of Optimized Red-Shifted Cameleons for Imaging ER Ca^2+^ Dynamics Using GFP/RFP FRET Pairs

**Figure 2:**
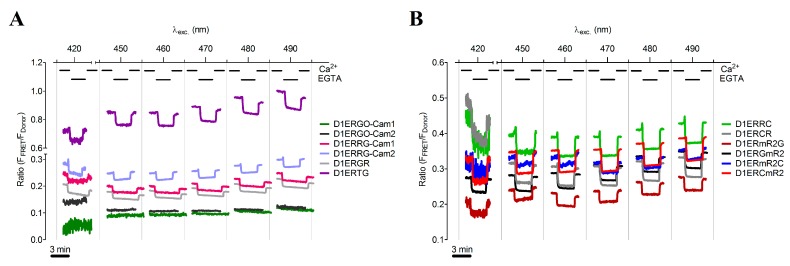
Functional evaluation of red-shifted cameleons in the ER. Permeabilized HeLa cells transfected with individual red-shifted sensors targeted to the ER were recorded in an extracellular Ca^2+^-free solution and in a 2 mM Ca^2+^ containing environment, respectively. Representative curves showing FRET signals of each indicator excited at 420, 450, 460, 470, 480 or 490 nm and emissions were collected from either (**A**) D1ERGO-Cam1 (dark grey curve), D1ERGO-Cam2 (green curve), D1ERRG-Cam1 (pink curve), D1ERRG-Cam2 (purple curve), D1ERGR (light grey curve), D1ERTG (violet curve) or (**B**) D1ERRC (light green curve), D1ERCR (grey curve), D1ERGmR2 (black curve), D1ERmR2G (dark red curve), D1ERmR2C (blue curve) or D1ERCmR2 (red curve).

**Figure 3:**
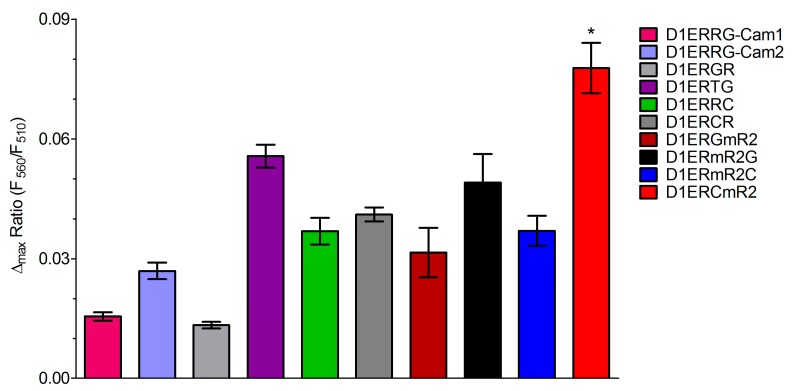
Column statistics of maximal ER Ca^2+^-release in HeLa cells transfected with individual GFP/RFP-based cameleons. Bars representing FRET change upon treatment with 100 µM histamine and 15 µM BHQ detected by D1ERRG-Cam1 (pink bar, n = 13), D1ERRG-Cam2 (purple bar, n = 10), D1ERGR (light grey bar, n = 4), D1ERTG (violet bar, n = 7), D1ERRC (light green bar, n = 10), D1ERCR (grey bar, n = 4), D1ERGmR2 (black bar, n = 11), D1ERmR2G (dark red bar, n = 6), D1ERmR2C (blue bar, n = 11) or D1ERCmR2 (red bar, n = 7). * *P* < 0.05 for D1ERCmR2 *vs*. all other indicators**.**

Under consideration of the fluorescent properties of various FPs, we selected several green FRET-donor and red FRET-acceptor proteins for the generation of novel functional red-shifted ER targeted cameleons (Table S2). For this purpose, we also included the green Clover and the red mRuby FP that had been recently used as an optimized FRET pair for the development of red-shifted genetically encoded probes for kinase activities and the membrane potential [41] Moreover the N- or C-terminal orientation of the FRET-donor and -acceptor FPs within ER targeted cameleons was considered as a relevant determinant of the sensors’ properties (ER localization and Ca^2+^ sensitivity). In total, we generated and tested 10 different ER targeted GFP/RFP-based cameleons that differ either in the FP-FRET pair or the orientation of FPs within the construct (C- or N-terminal location, Table 1). To test the properties of the novel red-shifted ER targeted cameleons, HeLa cells expressing the probes were imaged. During imaging at different excitation wavelengths ranging from 400 to 490 nm, ER Ca^2+^ levels were manipulated by cell treatment with digitonin/ionomycin in the presence and absence of Ca^2+^ in the experimental buffer (Figure 2A,B). Out of these spectral scans, the signal to noise ratio (SNR) for each sensor at each excitation wavelength was calculated to evaluate the best working red-shifted cameleon in the ER under these conditions (Table 2). All the GFP/RFP-based sensors were suitable to detect changes of [Ca^2+^]_ER_ (Figure 2). D1ERTG (Table 1 and Figure 2A), which was built of the bright tandem dimer Tomato as the FRET acceptor and EGFP as the FRET donor at the C-terminal end, appeared with the highest basal FRET ratio (Figure 2A), while the SNR was rather low (Table 2). Interestingly, the orientation of the FPs within the EGFP/tagRFP-consisting cameleons influenced the SNR significantly. If the tagRFP FRET acceptor was on the N-terminus of the ER targeted cameleon, better signals could be obtained (Figure 2A and Table 2). However, the cameleon with Clover on its N-terminal end and mRuby2 on its C-terminal end, named D1ERCmR2, showed the best performance with the highest SNR (Table 1 and Table 2; Figure 2B). This finding is in line with the original report about the usage of the Clover/mRuby FRET pair in genetically encoded probes [41] These are reporters of kinase activity (Camuiα-CR, AKAR-CR), small GTPase activity (Raichu-CR) and transmembrane voltage (VSFP-CR) [41]. Within all these indicators, the standard FRET pair CFP/YFP has been replaced by Clover as donor at the N-terminal end and by mRuby2 as FRET acceptor at the C-terminal end. Moreover, Clover and mRuby2 were characterized as the brightest green and red FPs exhibiting the highest Förster radius of any ratiometric FRET pair to date. We also tested if the orientation of Clover and mRuby in ER targeted cameleons impact the performance of the probe. In this case, the exchange of the FPs resulting in the construct D1ERmR2C (with mRuby2 on the N-terminus and Clover on the C-terminus) dramatically worsened the respective signal (Figure 2B; compare red curve with blue curve and respective SNR values in Table 2). Similar results were also found in intact HeLa cells that were treated for several minutes with histamine and the reversible sarcoplasmic/endoplasmic reticulum calcium ATPase (SERCA) inhibitor 2,5-di-tert-butylhydroquinone (BHQ) to transiently deplete the ER Ca^2+^ content (Figure 3). In line with the data obtained from the spectral scans, D1ERCmR2 showed a significantly higher delta FRET ratio signal in comparison with all other ER targeted probes in intact cells under these conditions (Figure 3). These findings indicate that in GFP/RFP-based cameleons that contain D1 as the Ca^2+^ sensing domain keep the high *K*_d_ of approximately 200 µM and are, hence, suitable to monitor Ca^2+^ dynamics of the ER. However, the origin and orientation of FPs forming the FRET pair within ER targeted cameleons have a huge impact on FRET ratio signals in response to ER Ca^2+^ mobilization.

**Table 1:** List of GFP/RFP FRET-based cameleons targeted to the ER.

Sensor Name	Schematic Overview
D1ERRG-Cam1	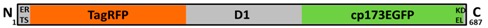
D1ERRG-Cam2	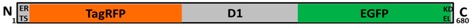
D1ERGR	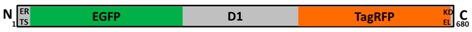
D1ERTG	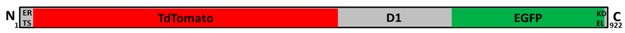
D1ERRC	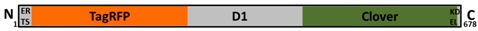
D1ERCR	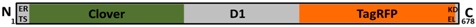
D1ERmR2G	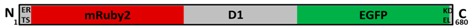
D1ERGmR2	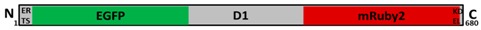
D1ERmR2C	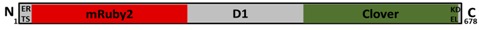
D1ERCmR2	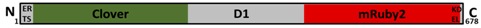

**Table 2:** Signal to noise ratios of various ER targeted red-shifted cameleons at stepwise increased excitation wavelengths. Optimum excitations for individual sensors resulting in highest SNR are highlighted.

Sensor	400	410	420	430	440	450	460	470	480	490
D1ERGO-Cam1	0.39	1.90	1.91	4.00	4.41	5.04	6.88	11.26	**12.14**	6.03
D1ERGO-Cam2	2.12	2.87	3.43	5.72	6.43	10.07	18.49	28.21	30.96	**32.32**
D1ERRG-Cam1	19.51	25.93	32.90	28.92	41.51	29.91	47.34	**57.18**	56.88	48.52
D1ERRG-Cam2	29.96	43.93	57.30	84.84	72.16	84.43	70.16	**77.38**	74.34	66.53
D1ERGR	4.90	6.99	7.53	12.17	16.09	27.44	40.84	55.94	**56.59**	41.48
D1ERTG	15.08	19.64	37.10	25.63	29.60	38.43	**44.83**	42.29	41.86	37.52
D1ERRC	2.47	3.57	5.06	8.33	14.23	23.26	47.45	81.02	**92.87**	92.57
D1ERCR	6.71	5.79	6.49	12.40	19.04	42.11	77.67	**142.01**	133.05	123.24
D1ERmR2G	9.84	13.56	17.92	22.72	25.01	50.53	82.87	107.33	**115.81**	107.91
D1ERGmR2	9.36	10.94	15.66	18.13	26.91	44.56	58.36	**70.51**	63.02	60.20
D1ERmR2C	1.99	2.26	2.74	3.68	5.21	11.83	18.93	30.48	31.09	**40.35**
D1ERCmR2	16.70	15.85	17.65	33.00	51.42	85.53	151.17	246.90	**263.13**	246.64

### 3.3. D1ERCmR2 Has a K_d_ of 200 µM in Situ and Respond to Ca^2+^ over a Broad Range

**Figure 4:**
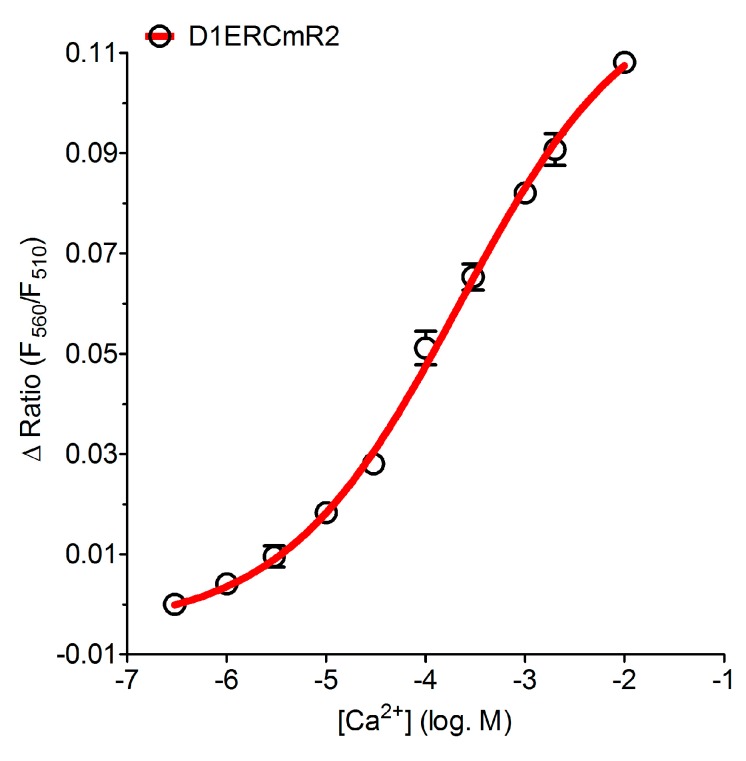
Determination of the *K*_d_ in permeabilized HeLa cells expressing D1ERCmR2. [Ca^2+^] was titrated to quantify the FRET ratio in percentage upon Ca^2+^-binding within D1ERCmR2 at 1 µM (n = 21), 3 µM (n = 21), 10 µM (n = 37), 30 µM (n = 37), 100 µM (n = 26), 300 µM (n = 43), 1 mM (n = 35), 2 mM (n = 17) and at 10 mM (n = 18).

In order to verify whether or not GFP/RFP-based ER targeted cameleons have a *K*_d_ that is suitable for imaging ER Ca^2+^ dynamics we determined the *K*_d_ of D1ERCmR2 *in situ*. Therefore we used ionomycin and digitonin permebilized HeLa cells and titrated Ca^2+^ at various concentrations ranging from 1 µM to 10 mM. These experiments revealed a *K*_d_ of 215.9 (150.7–309.4) µM for D1ERCmR2 (Figure 4). Originally *in vitro* studies of D1ER revealed 2 *K*_d_s, one at 0.8 µM and another at 60 µM [17,34]. However, in *in situ* experiments also using HeLa cells, the *K*_d_ of this probe was determined to be 220 µM [42] which is in agreement with the *K*_d_ that was found in this study for the red-shifted D1ERCmR2. Notably, the Hill slope of the Ca^2+^ concentration response curve of D1ERCmR2 was found to be 0.50 (0.39–0.60), which indicates that the red-shifted cameleon allows better detecting changes of ER Ca^2+^ over a broad range.

### 3.4. Dual Recordings of [Ca^2+^]_ER_ and [Ca^2+^]_Cyto_ in Single Fura-2 Loaded Cells Expressing D1ERCmR2

**Figure 5:**
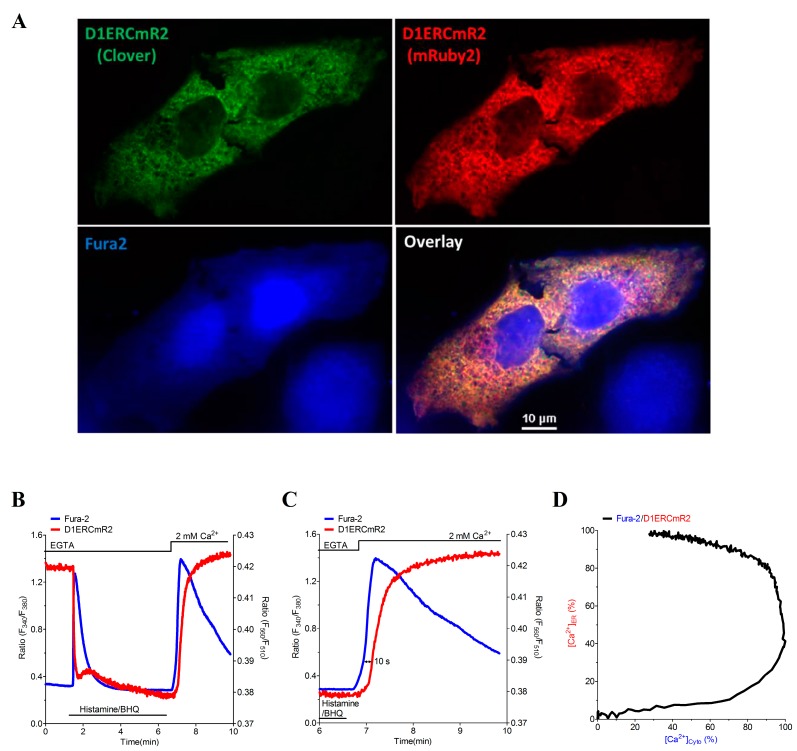
Dual visualization of fura-2 and D1ERCmR2 in single individual HeLa cells. (**A**) Confocal images of D1ERCmR2 expressing and fura-2 loaded HeLa cells. Subcellular structures of D1ERCmR2 were either illuminated at 473 or 515 nm and emissions were recorded at 525 (Clover, upper left panel) or 570 nm (mRuby2, upper right panel), respectively. Fura-2 was excited at 405 and emitted light was imaged at 460 nm (lower left panel). Images overlaid (lower right panel); (**B**) Representative curves of cytosolic and ER [Ca^2+^] in a single HeLa cell treated with 100 µM Histamine and 15 µM BHQ in a nominal Ca^2+^ free buffer and subsequent readdition of 2 mM Ca^2+^; (**C**) Zoom into event of SOCE reveals a delayed ER Ca^2+^ refilling; (**D**) Spatiotemporal correlation of [Ca^2+^]_Cyto_ and [Ca^2+^]_ER_ during SOCE in percentage of the maximum increase shown in panel C.

Because of the different spectral properties of the red-shifted ER targeted cameleon D1ERCmR2 compared with fura-2 (Fura2), the combination of both Ca^2+^ sensors allows simultaneous recordings of [Ca^2+^]_ER_ and [Ca^2+^]_Cyto_ in single individual cells. As expected, imaging of fura-2 loaded HeLa cells that express D1ERCmR2 revealed no fluorescence overlap of the respective fluorescence signals (Figure 5A). Cell treatment with a combination of the IP_3_-generating agonist histamine and the SERCA inhibitor BHQ instantly reduced the FRET ratio signal of D1ERCmR2 and increased the fura-2 ratio (Figure 5B and Video S1), indicating that under these conditions ER Ca^2+^ is mobilized quickly, resulting in a fast elevation of the cytosolic Ca^2+^ concentration. As the cell was stimulated in the absence of extracellular Ca^2+^, the cytosolic Ca^2+^ elevation turned back to basal levels within one minute. The subsequent addition of Ca^2+^ upon the removal of histamine and BHQ resulted in a transient rise of [Ca^2+^]_Cyto_ due to SOCE. The simultaneous recording of ER Ca^2+^ revealed that during SOCE, the ER gets refilled with a delay of almost 10 s (Figure 5C). Moreover, this approach highlights that the cytosolic Ca^2+^ elevation stops when approximately 40% of the ER Ca^2+^ content is restored (Figure 5C,D). While these data are in line with the kinetics of STIM1 oligomerization and punctae formation [43] further experiments are necessary to investigate the temporal correlation of SOCE activation and termination with respective ER Ca^2+^ dynamics.

**Figure 6:**
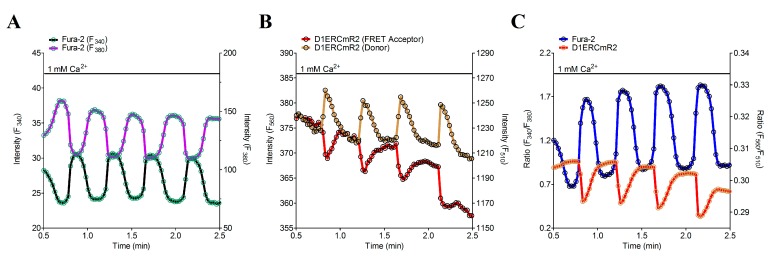
Time course of [Ca^2+^]_Cyto_ and [Ca^2+^]_ER_ in a single HEK E2 cell visualizing oscillatory traces in 1 mM Ca^2+^. (**A**) Single emission curves of fura-2 either excited at 340 nm (black curve) or 380 nm (blue curve); (**B**) Donor and FRET acceptor fluorescences of D1ERCmR2 at 560 nm (red curve) or 510 nm (green curve), respectively; (**C**) Overlay of ratio curves from fura-2 (blue curve) or D1ERCmR2 (red curve).

The approach of dual recordings of [Ca^2+^]_Cyto_ and [Ca^2+^]_ER_ was further tested using HEK-293 cells stably expressing the ryanodine receptor 2 (RYR2). RYR2 is referred to as cardiac Ca^2+^ release channel that makes HEK-293 cells (HEK E2) inducible to extracellular Ca^2+^ [35,44]. Ca^2+^ addition is known to activate store overload induced Ca^2+^ release (SOICR) in the HEK E2 clone. SOICR is frequently used to characterize the RYR2 channel activity using these heterologous expression system [35,44]. In simultaneous measurements using D1ERCmR2 as a sensor for [Ca^2+^]_ER_ and fura-2 as an indicator for [Ca^2+^]_Cyto_, we observed cytosolic and ER Ca^2+^ oscillations in the HEK E2 clone (Figure 6A–C). This data show that the on and off kinetics of D1ERCmR2 are as fast to follow such SOICR-dependent oscillations and that this approach can be used to investigate the Ca^2+^-dependent activation of the RYR2 in more detail. Particularly, the simultaneous recording of [Ca^2+^]_Cyto_ and [Ca^2+^]_ER_ in this cell model should allow determining the ratio of [Ca^2+^]_Cyto_/[Ca^2+^]_ER_ upon clusters of RyR2 get activated. However, fura-2 as a Ca^2+^ buffer might affect the frequency of SOICR under these conditions.

## 4. Conclusions

In this study, we show that GFP/OFP-based cameleons are impractical to monitor ER Ca^2+^ signals, while red-shifted ER targeted cameleons that consist of a GFP/RFP FRET pair are suitable to image ER Ca^2+^ dynamics in single cells. A cameleon, referred to as D1ERCmR2, which is based on the Clover/mRuby FRET pair, was shown to have a good signal to noise ratio, optimal *K*_d_ and Hill slope to record dynamic changes of ER Ca^2+^ in mammalian cells. Moreover, this red-shifted probe can be used simultaneously with fura-2 in order to correlate [Ca^2+^]_ER_ and [Ca^2+^]_Cyto_ in single individual cells. With this work, we expanded the palette of red-shifted organelle targeted Ca^2+^ probes that show less phototoxicity and should be compatible with most of the existing confocal and wide-field fluorescence imaging systems.

## Supplementary Materials

### Video S1

Simultaneous video sequences of D1ERCmR2 and fura-2 during Ca^2+^ entry SOCE in a single HeLa cell. Original recordings and curves of donor (green upper left panel and green curve) and acceptor (red lower left panel and red curve) from D1ERCmR2 as well as the fura-2 signals at 340 nm excitation (blue upper right panel and blue curve) and at 380 nm (yellow lower right panel and yellow curve) were aligned to present the recorded time-lapse. The ratio changes of D1ERCmR2 (lower red curve) and of fura-2 (lower white curve) were calculated from the upper sequences and displayed in percentage of maximum response.

Supplementary materials can be accessed at: http://www.mdpi.com/1424-8220/15/6/13052/s1.
