# Supplementary material for: Generation of Red-Shifted Cameleons for Imaging Ca2+ Dynamics of the Endoplasmic Reticulum
Source: Sensors (Basel). 2015 Jun 4;15(6):13052–68. doi: 10.3390/s150613052 (PMC4507692; doi:10.3390/s150613052)
Supplement: Supplementary file 1 [file sensors-15-13052-s001.pdf]

OPEN ACCESS
sensors
ISSN 1424-8220
www.mdpi.com/journal/sensors

Supplementary Information

Generation of Red-Shifted Cameleons for Imaging Ca^{2+} Dynamics of the Endoplasmic Reticulum. *Sensors* 2015, *15*, 13052-13068

Markus Waldeck-Weiermair *, Helmut Bischof, Sandra Blass, Andras T. Deak, Christiane Klec, Thomas Graier, Clara Roller, Rene Rost, Emrah Eroglu, Benjamin Gottschalk, Nicole A. Hofmann, Wolfgang F. Graier and Roland Malli

Institute of Molecular Biology and Biochemistry, Centre of Molecular Medicine, Medical University of Graz, Harrachgasse 21, 8010 Graz, Austria;
E-Mails: bischof.helmut@gmail.com (H.B.); sandra.blass@medunigraz.at (S.B.); andras.deak@medunigraz.at; (A.T.D.); christiane.klec@medunigraz.at (C.K.); thomas.graier@medunigraz.at (T.G.); clara.roller@gmx.at (C.R.); rene.rost@medunigraz.at (R.R.); emrah.eroglu@medunigraz.at (E.E.); benjamin.gottschalk@medunigraz.at (B.G.); nicole.hofmann@medunigraz.at (N.A.H.); wolfgang.graier@medunigraz.at (W.F.G.); roland.malli@medunigraz.at (R.M.)

* Author to whom correspondence should be addressed: markus.weiermair@medunigraz.at; Tel.: +43-316-380-7562; Fax: +43-316-380-9615.

Table S1. List of primers and restriction sites for engineering red-shifted cameleons.

Constructs	Gene	Orientation	Primer Pairs	Restriction Sites
All ER targeted	ER Signal sequence of Calreticulin	N-terminal of all ER targeted sensors	Sense strand: 5′-CTAGCATGCTGCTGCCCGTCCCCCTG CTGCTGGGCCTGCTGGGCGCCGCCGCCGAC-3′ Antisense strand: 5′-GTACGACGACGGGCAGGGGGACGA CGACCCGGACGACCCGCGGCGGCGGCTGAGCT-3′	NheI/XhoI
D1ERGR, D1ERGmR2	EGFP	N-terminal	Forward: 5′-AATCTCGAGTATGGTGAGCAAGGGCGAGGA-3′ Reverse: 5′-GCCATCGATCTTGTACAGCTCGTCCATGC-3′	XhoI/ClaI
D1ERGO-Cam2, D1ERRG-Cam2, D1ERTG, D1ERmR2G	$EGFP_{KDEL}$	C-terminal	Forward: 5′-CGGGAATTCATGGTGAGCAAGGGCGAGGA-3′ Reverse: 5′-GCAAGCTTACAGCTCGTCCTTGTACAGCTCGTCCATGC-3′	EcoRI/HindIII
D1ERRG-Cam1, D1ERRG-Cam2, D1ERRC	TagRFP	N-terminal	Forward: 5′-GGCTCGAGTATGGTGTCTAAGGGCGAAGAG-3′ Reverse: 5′-GGATCGATATTAAGTTTGTGCCCCAGTTTGC-3′	XhoI/ClaI
D1ERGR, D1ERCR	$TagRFP_{KDEL}$	C-terminal	Forward: 5′-TCGGAATTCATGGTGTCTAAGGGCGAAGAG-3′ Reverse: 5′-GCGAAGCTTACAGCTCGTCCTTATTAAGTT TGTGCCCCAG-3′	EcoRI/HindIII
D1ERTG	tdTomato	N-terminal	Forward: 5′-AATCTCGAGTATGGTGAGCAAGGGCGAGGA-3′ Reverse: 5′-CGAATCGATCTTGTACAGCTCGTCCATGC-3′	XhoI/ClaI
D1ERCR, D1ERCmR2, D1CmR2, mtD1CmR2	Clover	N-terminal	Forward: 5′-AATCTCGAGTATGGTGAGCAAGGGCGAGGA-3′ Reverse: 5′-AGAATCGATAGCTCGAGATCTGAGTCCGGC-3′	XhoI/ClaI
D1ERRC, D1ERmR2C	$Clover_{KDEL}$	C-terminal	Forward: 5′-AGGGAATTCATGGTGAGCAAGGGCGAGGAG-3′ Reverse: 5′-AGCAAGCTTACAATTCGTCCTTAGCTCGAGATCTGAGTCCGGC-3′	EcoRI/HindIII
D1ERmR2G, D1ERmR2C	mRuby2	N-terminal	Forward: 5′-ACTCTCGAGTATGGTGTCTAAGGGCGAAGAGC-3′ Reverse: 5′-AGCATCGATCTTGTACAGCTCGTCCATCCCAC-3′	XhoI/ClaI
D1ERGmR2, D1ERCmR2	$mRuby2_{KDEL}$	C-terminal	Forward: 5′-AGCGAATTCATGGTGTCTAAGGGCGAAGAGC-3′ Reverse: 5′-GACAAGCTTACAGCTCGTCCTTCTTGTACA GCTCGTCCATCC-3′	EcoRI/HindIII
D1CmR2, mtD1CmR2	mRuby2	C-terminal	Forward: 5′-AGCGAATTCATGGTGTCTAAGGGCGAAGAGC-3′ Reverse: 5′-GCCAAGCTTACTTGTACAGCTCGTCCATCC-3′	EcoRI/HindIII

Table S2. Properties of fluorescent proteins.

Colour	Protein	Length	λ_{Exc}(nm)	λ_{Em}(nm)	Brightness *	Photostability	pKA	Ref.
**Cyan**	ECFP	238	435	478	13	64	5.5	[1]
**Yellow**	Citrine	238	516	529	59	49	5.7	[2]
**Green**	EGFP	238	488	507	34	174	6.0	[2]
	Clover	232	505	515	84	50	6.1	[3]
**Orange**	mKO	217	548	559	31	122	5.0	[1]
**Red**	TagRFP	237	555	515	48	48	3.8	[4]
	Td-tomato	476	554	581	95	98	4.7	[2,4]
	mRuby2	238	559	600	43	123	5.3	[3]

* Brightness: Molar Extinction Coefficient × Fluorescence Quantum Yield/1000.

References

1. Karasawa, S.; Araki, T.; Nagai, T.; Mizuno, H.; Miyawaki, A. Cyan-Emitting and Orange-Emitting Fluorescent Proteins as a Donor/Acceptor Pair for Fluorescence Resonance Energy Transfer. *Biochem. J.* **2004**, *381*, 307–312.
2. Shaner, N.C.; Steinbach, P.A.; Tsien, R.Y. A Guide to Choosing Fluorescent Proteins. *Nat. Methods* **2005**, *2*, 905–909.
3. Lam, A.J.; St-Pierre, F.; Gong, Y.; Marshall, J.D.; Cranfill, P.J.; Baird, M.A.; McKeown, M.R.; Wiedenmann, J.; Davidson, M.W.; Schnitzer, M.J.; *et al.* Improving FRET Dynamic Range with Bright Green and Red Fluorescent Proteins. *Nat. Methods* **2012**, *9*, 1005–1012.
4. Shaner, N.C.; Lin, M.Z.; McKeown, M.R.; Steinbach, P.A.; Hazelwood, K.L.; Davidson, M.W.; Tsien, R.Y. Improving the Photostability of Bright Monomeric Orange and Red Fluorescent Proteins. *Nat. Methods* **2008**, *5*, 545–551.

© 2015 by the authors; licensee MDPI, Basel, Switzerland. This article is an open access article distributed under the terms and conditions of the Creative Commons Attribution license (http://creativecommons.org/licenses/by/4.0/).
