# Supplementary figures and images for: Generation of Red-Shifted Cameleons for Imaging Ca2+ Dynamics of the Endoplasmic Reticulum
Source: Sensors (Basel). 2015 Jun 4;15(6):13052–68. doi: 10.3390/s150613052 (PMC4507692; doi:10.3390/s150613052)

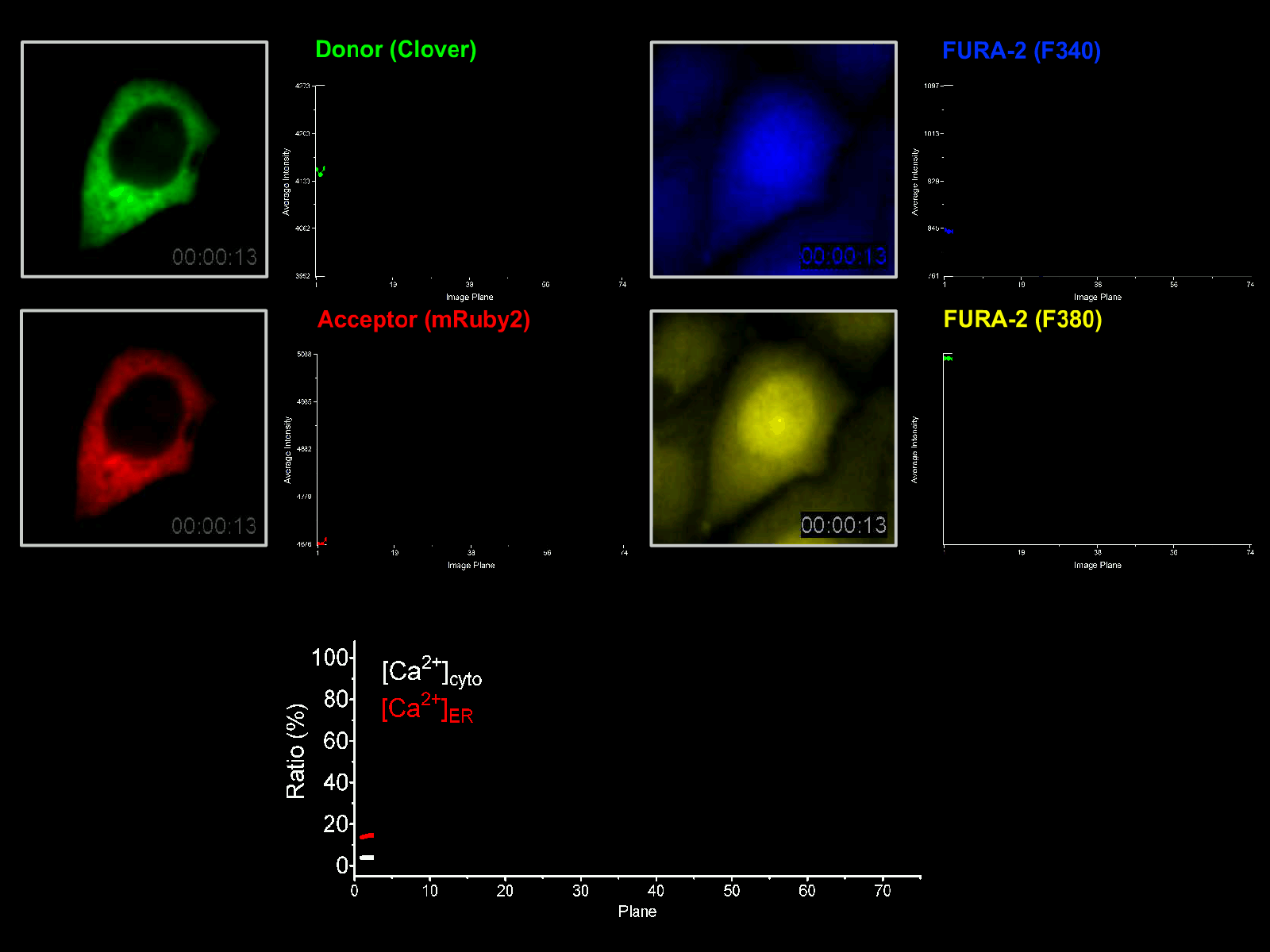

Supplement: Supplementary file 2 [file sensors-15-13052-s002.gif]
